# Comparative Study of the Effect of Doping ZnTiO_3_ with Rare Earths (La and Ce) on the Adsorption and Photodegradation of Cyanide in Aqueous Systems

**DOI:** 10.3390/ijms24043780

**Published:** 2023-02-14

**Authors:** Ximena Jaramillo-Fierro, Guisella Cuenca, John Ramón

**Affiliations:** 1 Departamento de Química, Facultad de Ciencias Exactas y Naturales, Universidad Técnica Particular de Loja, San Cayetano Alto, Loja 1101608, Ecuador; 2 Ingeniería Química, Facultad de Ciencias Exactas y Naturales, Universidad Técnica Particular de Loja, San Cayetano Alto, Loja 1101608, Ecuador

**Keywords:** adsorption, photocatalysis, cyanide, zinc titanate, lanthanides, nanoparticles

## Abstract

Cyanide is a highly toxic compound that can pose serious health problems to both humans and aquatic organisms. Therefore, the present comparative study focuses on the removal of total cyanide from aqueous solutions by photocatalytic adsorption and degradation methods using ZnTiO_3_ (ZTO), La/ZnTiO_3_ (La/ZTO), and Ce/ZnTiO_3_ (Ce/ZTO). The nanoparticles were synthesized by the sol-gel method and characterized by X-ray powder diffractometry (XRD), Scanning electron microscopy (SEM), Energy-dispersive X-ray spectroscopy (EDS), Diffuse reflectance spectroscopy (DRS), and Specific surface area (SSA). The adsorption equilibrium data were fitted to the Langmuir and Freundlich isotherm models. Adsorption kinetics were also evaluated using the pseudo-first-order and pseudo-second-order models and the intraparticle diffusion model. Likewise, the photodegradation of cyanide under simulated sunlight was investigated and the reusability of the synthesized nanoparticles for cyanide removal in aqueous systems was determined. The results demonstrated the effectiveness of doping with lanthanum (La) and cerium (Ce) to improve the adsorbent and photocatalytic properties of ZTO. In general, La/ZTO showed the maximum percentage of total cyanide removal (99.0%) followed by Ce/ZTO (97.0%) and ZTO (93.6%). Finally, based on the evidence of this study, a mechanism for the removal of total cyanide from aqueous solutions using the synthesized nanoparticles was proposed.

## 1. Introduction

Cyanide is commonly found as a contaminant in wastewater from various industries [1]. Despite its toxicity, cyanide is also found in a wide variety of life forms, including in the animal kingdom. Cyanide is a compound that occurs in nature in the form of cyanide, cyanate, and nitrile compounds, among others. Free cyanide is made up of the cyanide ion (CN^−^) and hydrogen cyanide (HCN). This compound is highly dangerous and can even cause death. Due to the potential hazard associated with cyanide, it is of great importance to control and remediate cyanide-contaminated water prior to its discharge into the environment [2].

Currently, cyanide-containing wastewater is treated by methods of chemical and electrolytic oxidation, ion exchange, acidification, reverse osmosis, flotation, precipitation, and thermal hydrolysis; however, all of these are expensive methods, and they require dangerous chemical reagents, specialized equipment, maintenance, and additional levels of detoxification [3,4]. Compared to these processes, adsorption and photocatalysis are promising methods for the treatment of cyanide-contaminated wastewater, due to their high removal capacity, simplicity in their design, ease, and low cost of operation, in addition to producing cyanide-free effluents, which can be reused [5]. Adsorption is a process that can be carried out by different mechanisms, including electrostatic interaction, complex formation, or ion exchange between the adsorbate and the adsorbent. Heterogeneous photocatalysis is an advanced oxidation process (POA) that allows the complete mineralization of several compounds, through a sequence of oxidation-reduction reactions that begin with the photoactivation of the catalyst, either with natural light or artificial [6,7,8,9,10,11].

The adsorption and photodegradation capacity of materials is usually influenced by several factors that depend on the properties of the material, as well as the operating conditions [12]. Investigation of these parameters provides valuable information for the design of materials and processes for the efficient removal of a specific contaminant [13]. For the adsorption and photocatalysis processes to be efficient, the materials used must have a large specific surface area, several active sites on their surfaces, chemical stability, a high adsorption capacity, a high oxidative capacity, and a lack of toxicity. In addition, they must be friendly to the environment, highly efficient, economical, regenerable, and easily affordable [14].

Recently, various materials have been tested for the removal of cyanide compounds, such as activated carbon, clays, zeolites, and metal oxides, among others [15,16,17,18,19,20,21,22]; however, no previous studies on the use of the ZnTiO_3_ semiconductor in the removal of these contaminants were found. This polar oxide is formed by Ti^4+^ (3d^0^) and Zn^2+^ (3d^10^), which present a strong coulombic repulsion towards each other [23,24], which allows ZnTiO_3_ to be used as a material with ferroelectric, nonlinear optical and piezoelectric properties and as a photocatalyst in environmental remediation processes [25,26,27,28,29,30,31,32]. Like other semiconductors, ZnTiO_3_ can be doped as an effective proposition to alter its intrinsic electronic structure and improve its physicochemical properties. Doping allows a reduction in the bandgap energy of the semiconductor and therefore improves its sensitivity to visible light, also allowing a better separation of the photogenerated charges and therefore a greater quantum efficiency in photochemical processes [33,34].

Semiconductor doping with lanthanum (La) and cerium (Ce) has recently received a lot of attention for the preparation of versatile photocatalysts in which the concentration of the doping element does not exceed 1–2% by weight of the photocatalyst [35,36,37,38,39,40,41,42,43,44]. Both lanthanides are rare earth elements widely investigated to improve the photocatalytic activity of semiconductors, in a wide range of optical absorption [45,46,47,48,49]. These elements are used as efficient dopants of materials for various applications due to their chemical, electronic, and optical characteristics that result from the transition of their 4f electrons [50,51]. According to the literature, lanthanum has an oxidation state La^3+^ (5d^0^ 6s^0^) and forms the oxide La_2_O_3_, while cerium has two oxidation states, Ce^3+^ (4f^1^ 5d^0^) and Ce^4+^ (4f^0^ 5d^0^), and, under conditions of reducing and oxidizing, Ce can switch between the oxides Ce_2_O_3_ and CeO_2_. These oxides promote different optical properties because they have different electronic configurations [44].

Evidence from the literature shows that doping semiconductor oxides with La and Ce can reduce crystal growth [44] and increase the specific surface area and concentration of surface hydroxyl (−OH) groups, as well as improve optical properties of these semiconductors due to the increased concentration of oxygen vacancies. Furthermore, they can enhance the chemical adsorption of organic substrates on the semiconductor surface, which also enhances their photocatalytic efficiency for various applications [52,53,54,55,56,57,58,59].

In the literature, there are many reports on the adsorption removal of NaCN, KCN, and gold cyanide complexes, but to the best of our knowledge, there are no reports mentioning the adsorption and photodegradation of cyanide species by using the semiconductors ZnTiO_3_, La/ZnTiO_3_, and Ce/ZnTiO_3_; thus, the behavior of these semiconductors in the adsorption and photodegradation processes of cyanide species compounds is not clear. Therefore, the objective of this comparative study is to evaluate the effect of La and Ce ions on the adsorbent and photocatalytic capacity of ZnTiO_3_ for the removal of total cyanide in aqueous solutions. Cyanide adsorption and photodegradation in aqueous solutions were determined in batch experiments. The amount of total cyanide (HCN + CN^−^) residual in the solutions was determined by UV–visible spectrophotometry using the picrate alkaline method. The adsorption capacity of the synthesized compounds was evaluated by varying the composition of the doping element, the pH of the solutions, the concentration of the adsorbent in the solution, and the contact time, while the photocatalytic activity was determined under simulated solar irradiation (λ = 300–800 nm). The synthesized compounds were characterized by X-ray diffractometry (XRD), diffuse reflectance spectroscopy (DRS), scanning electron microscopy (SEM-EDX), and specific surface area (SSA).

## 2. Results

### 2.1. Characterization of the Nanoparticles

#### 2.1.1. XRD and SSA Analysis

**Figure 1:**
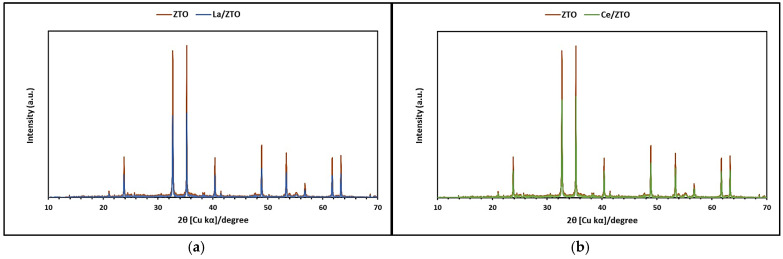
X-ray diffraction (XRD) pattern of the nanoparticles: (**a**) La/ZTO and (**b**) Ce/ZTO compared with ZTO.

Figure 1a,b show mixed oxides of titanium and zinc doped with lanthanum (La/ZTO) and cerium (Ce/ZTO), respectively, compared to the undoped mixed oxide (ZTO). These nanoparticles were synthesized at 500 °C with a TiO_2_:ZnO molar ratio of 1:1. The diffraction peaks of the nanoparticles at 2θ values of 23.92°, 32.79°, 35.31°, 40.45°, 48.93°, 53.44°, 56.82°, 61.79°, and 63.39° were assigned to the crystallographic planes (0 1 2), (1 0 4), (1 1 0), (1 1 3), (0 2 4), (1 1 6), (0 1 8), (2 1 4), and (3 0 0), respectively. The obtained nanoparticles were indexed to the hexagonal phase with unit cell parameters a = b = 5.08 Å and c = 13.93 Å and space group R-3(148) according to JCPDS standard card No. 00-015-0591. Due to the low concentration of the doping elements, the characteristic diffraction peaks reported in the literature were not reached for lanthanum oxide (2θ = 28° and 2θ = 39°) [60] or for cerium oxide (2θ = 28° and 2θ = 47°) [61].

The crystal sizes of ZTO, La/ZTO, and Ce/ZTO were calculated based on the main peak of the nanostructures using the well-known Scherrer equation shown below [62],

(1) $$A=\frac{K\lambda }{\beta \;\cos\theta }$$

where K = 0.89 and λ = 0.15406 nm correspond to the shape factor and the wavelength of the X-ray beam, respectively. The Bragg angle is represented by θ, while the full width at half peak height (FWHM) is represented by β. The average crystal sizes of the ZTO, La/ZTO, and Ce/ZTO nanomaterials were calculated at 41.35 (±1.27), 29.09 (±1.92), and 16.33 (±1.31) nm, respectively.

In the present study, the single-point BET (Brunauer-Emmet-Teller) method was used to determine the specific surface area (SSA) of the nanoparticles. For this, the sample was first degassed using a stream of ultrapure N_2_ gas. After degassing the sample at 200 °C for 2 h, a gas mixture of nitrogen and helium (30% nitrogen) was flowed over it. Then, a liquid nitrogen bath at −196 °C was placed to immerse the sample and N_2_ adsorption was recorded as a function of time. Once equilibrium was reached, the liquid nitrogen bath was removed and N_2_ desorption was recorded as a function of time until equilibrium was again reached. The liquid N_2_ adsorption–desorption plots for each of the nanoparticles are shown in Figure S1. The calculation of the total specific surface was carried out based on the amount of nitrogen desorbed and the mass of the sample evaluated. The SSA of ZTO was measured as 93 m^2^ g^−1^, which is slightly lower than that of the photocatalysts La/ZTO and Ce/ZTO with values of 128 and 112 m^2^ g^−1^, respectively. From these results, the specific effect of lanthanum and cerium on the inhibition of crystallite growth and the stabilization of ZnTiO_3_ mixed oxide, as well as the increase in the specific surface area (SSA), is suggested.

#### 2.1.2. SEM and EDS Analysis

**Figure 2:**
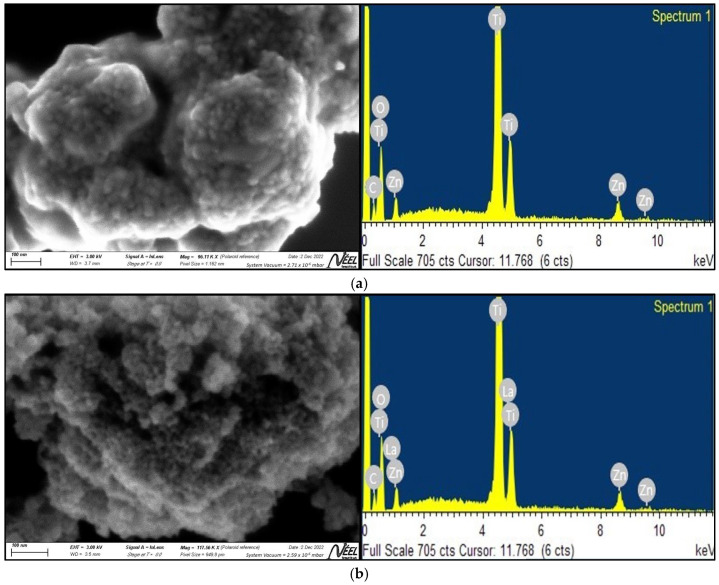

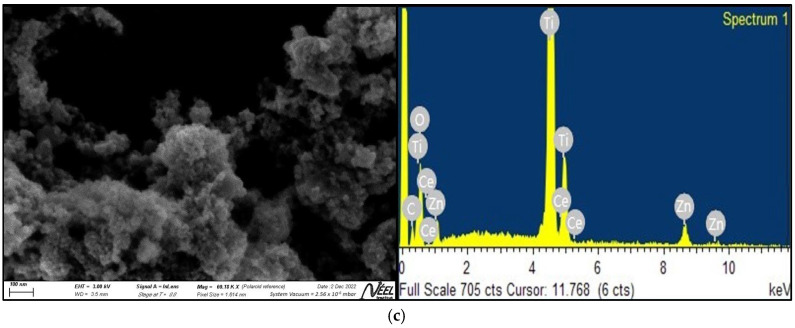
Scanning electron microscopy (SEM) images and energy-dispersive X-ray (EDX) spectra of (**a**) ZTO, (**b**) La/ZTO, and (**c**) Ce/ZTO.

Figure 2a–c display the images obtained by scanning electron microscopy (SEM) for the compounds ZTO, La/ZTO, and Ce/ZTO, respectively. In these figures, it is evident that the particles of the three compounds are nanometer in size, nearly spherical in shape, and quite agglomerated. The particles of the doped compounds were found to be smaller than the particles of the undoped ZTO compound. The mean particle size of ZTO was 94 nm, in contrast to the La/ZTO and Ce/ZTO particles, where the mean particle sizes were 59 and 67 nm, respectively. The results presented indicate that the rare earth doping elements lanthanum and cerium are effective in preventing crystallite growth and stabilizing the ZTO compound.

The presence of lanthanum and cerium in the ZnTiO_3_ mixed oxide was confirmed by energy-dispersive X-ray (EDX) spectroscopy. According to the EDX analysis shown in Figure 2, both lanthanum and cerium were efficiently incorporated into the ZTO nanoparticles. On average the proportion of these two rare earths in the ZTO nanostructure was around 1.19%, within the instrumental precision.

#### 2.1.3. Optical and Photoelectric Properties

**Figure 3:**
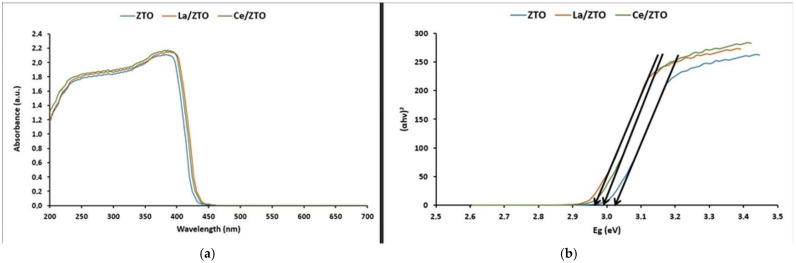
(**a**) UV-vis DRX and (**b**) plots of (αhv)^2^ vs. E_g_ of ZTO, La/ZTO, and Ce/ZTO.

XRD UV–visible (UV-vis) analysis was used to characterize the optical properties of the nanocomposites. The evaluation range was from 200 to 700 nm and the assay was carried out at room temperature. The comparative results of the assay are shown in Figure 3a. In this figure, it can be seen that La/ZTO and Ce/ZTO presented a slight improvement in the adsorption response in the visible range (around 400 nm) compared to ZTO. Due to this bathochromic shift, it is suggested that, compared to ZTO, the doped compounds have a better response to visible light and can prevent (e^−^/h^+^) pairs recombination, which in turn increases the catalyst lifetime. Plots of (αhv)^2^ vs. hv to calculate the direct bandgap energy (E_g_) are shown in Figure 3b.

Figure 3b shows the direct bandgap energy (E_g_) values obtained graphically [63] for ZTO (3.03 eV), La/ZTO (2.97 eV), and Ce/ZTO (2.97 eV). The direct E_g_ (eV) values shown on the energy axis were calculated using the expression [64]:

(2) $$E_{g}=\frac{1240}{\lambda }$$

where E_g_ is the bandgap energy in electronvolts (eV) and λ represents the lower cut-off wavelength in nanometers (nm).

### 2.2. Effect of Nanoparticles’ Composition

**Figure 4:**
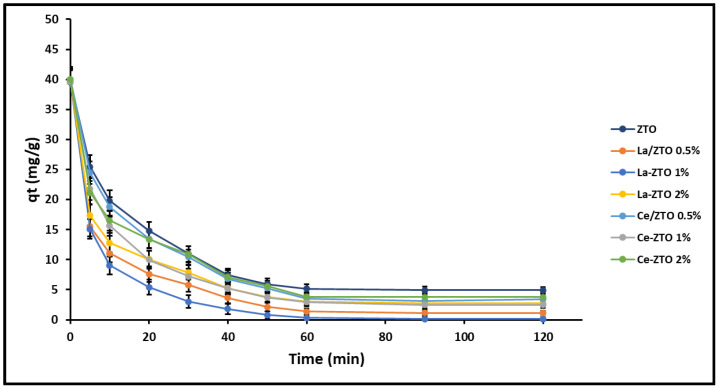
Cyanide adsorption capacity as a function of nanoparticles’ composition.

The effect of the composition of the nanoparticles on their cyanide removal capacity q_e_ (mg g^−1^) was first evaluated and the results are shown in Figure 4. The equilibrium time t_e_ (min) for all the nanoparticles was found at 90 min under the conditions of testing.

The results of the analysis of variance (ANOVA) performed at t_e_ (min) are summarized in Table 1. Values with different letters (a–f) in the same column are significantly different (*p* < 0.01). From these results, it is suggested that the best composition for doped compounds is with a content of 1% doping element (La or Ce). Therefore, the doped nanoparticles La/ZTO (1%) and Ce/ZTO (1%) were selected to perform cyanide removal tests and contrast their effectiveness with the undoped ZTO.

### 2.3. Adsorption Behaviour

#### 2.3.1. Effect of the pH of the Solution

**Figure 5:**
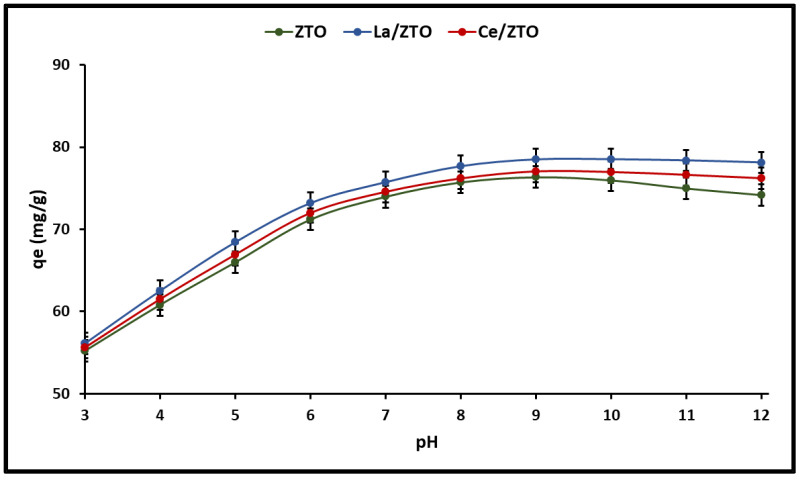
Cyanide adsorption capacity as a function of the pH of the solution.

Regarding the cyanide adsorption as a function of the pH of the solutions, the results of this study are shown in Figure 5. The total cyanide adsorption capacity (HCN + CN^−^) remained unchanged in the pH range of 9 to 12 for the doped nanoparticles (pH_PZC_ = 7.2). However, for pure ZTO (pH_PZC_ = 7.6) a slight decrease in total cyanide adsorption capacity could be observed in the same pH range. In this study, the pH_PZC_ of the materials was on average 7.4. This implies that at pH < pH_PZC_ = 7.4 the surface of the nanoparticles has a positive charge and a negative charge at pH > pH_PZC_ = 7.4. According to the literature, oxygen functional groups are important characteristics of adsorbents because they determine their surface area properties as well as their basic character [5]. Therefore, due to the average value of pH_PZC_ = 7.4 of the nanoparticles, it is suggested that they could have a slight dominance of basic groups.

The adsorption of ions from the solution is highly dependent on the pH of the solution, which affects the surface charge of the adsorbents and the degree of ionization and speciation of the substrate. Therefore, it is also important to consider the distribution of cyanide species in the solution due to the effect of pH (Equation (3)) for a better understanding of cyanide adsorption mechanisms [5].

(3) $$\mathrm{HCN}\;↔{\;\mathrm{CN}}^{-}\;\;\;\mathrm{pKa}=9.4$$

In aqueous solutions, inorganic cyanides (NaCN and KCN) can be easily hydrolyzed to generate hydrocyanic acid (HCN) whose pKa value is 9.4. Dissolved HCN is stable at pH < 9.4; however, at pH > 9.4 it dissociates to form CN^−^ ions [13], which have an important nucleophilic character [65]. Several authors have also reported that, at pH = 7, inorganic cyanides, being weak acids, can be easily hydrolyzed and form the species HCN and CN^−^ [66]. Furthermore, since HCN (25.6–26.6 °C) has a very low boiling point, it is likely that most of the HCN formed can evaporate from solutions at pH = 7.0. Consequently, several investigations on cyanide adsorption have been developed at pH values around 7.0 (pH < pKa), suggesting that the high adsorption of the HCN molecule in the adsorbent is based on interactions with Lewis acid sites [67,68,69,70]. Therefore, this study was carried out at a pH of 7.0.

#### 2.3.2. Adsorption Isotherm

**Figure 6:**
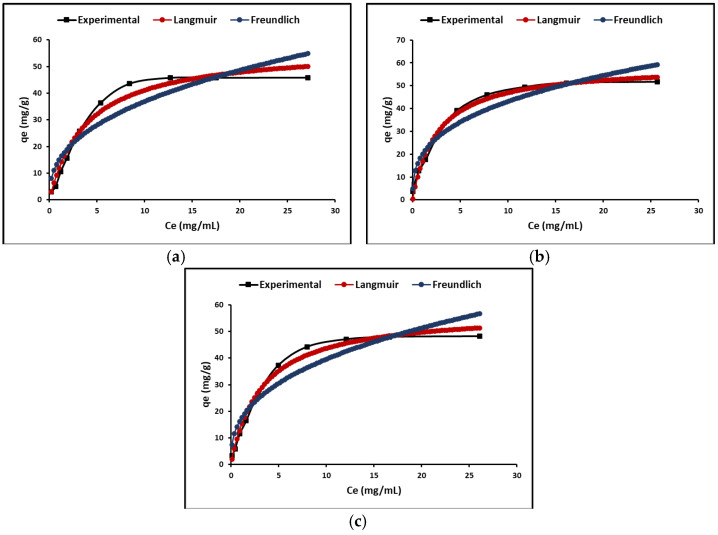
Adsorption isotherms of (**a**) ZTO, (**b**) La/ZTO, and (**c**) Ce/ZTO.

To understand adsorption phenomena, two types of models were developed in this study, equilibrium models (isotherms) based on initial concentration and kinetic models based on reaction time. The most common isotherms are Langmuir and Freundlich. According to Langmuir’s theory, adsorption occurs in a monolayer on a completely homogeneous surface with a finite number of identical and specific adsorption sites and with negligible interaction between molecules. The Freundlich model does not assume homogeneity in the energy of the sites on the surface; it assumes that the strongest binding sites are occupied first and that there is no limit on the maximum adsorption charge. Figure 6 shows the cyanide adsorption isotherms by the ZTO, La/ZTO, and Ce/ZTO nanoparticles. In this figure, it is evident that the Langmuir model is better than the Freundlich model to describe the adsorption behavior of all nanoparticles.

The calculated results of the Langmuir and Freundlich isotherm constants are given in Table 2. In this table, the separation factor R_L_ or equilibrium parameter is also reported. Since 0 < R_L_ < 1, it indicates favorable adsorption, while R_L_ > 1 indicates unfavorable adsorption, R_L_ = 0 indicates irreversible adsorption, and R_L_ = 1 indicates linear adsorption [71]; so, in all the cases analyzed here, the cyanide adsorption process is favorable since the calculated R_L_ value was less than one.

#### 2.3.3. Adsorption Kinetics

**Figure 7:**
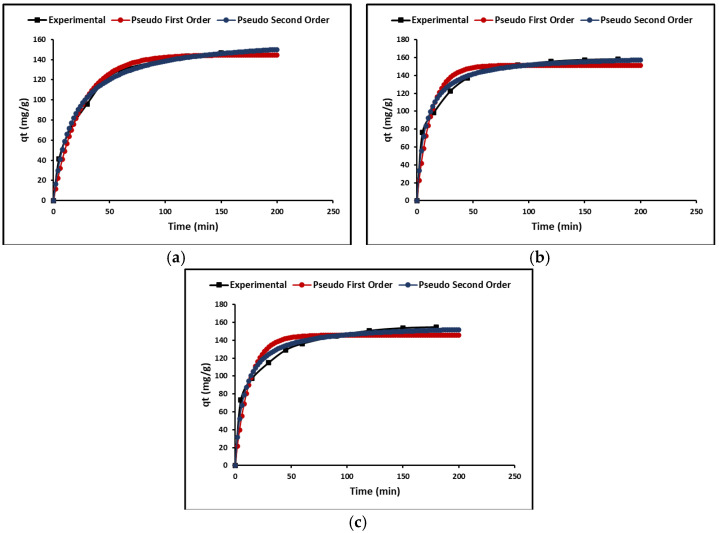
Adsorption kinetics of (**a**) ZTO, (**b**) La/ZTO, and (**c**) Ce/ZTO.

In this study, the kinetic models of pseudo-first-order, pseudo-second-order, and intraparticle diffusion models were used. Both the pseudo-first-order and the pseudo-second-order models show adsorption rate versus reaction time. To study these models, the graph between the amounts adsorbed, q_e_ (mg g^−1^), versus time, t (h), was plotted as shown in Figure 7. In this figure, it is evident that the adsorption is very fast initially, followed by the plateau stage. Table 3 shows that the correlation coefficient of the pseudo-second-order model is the highest, which could suggest a chemisorption process.

**Figure 8:**
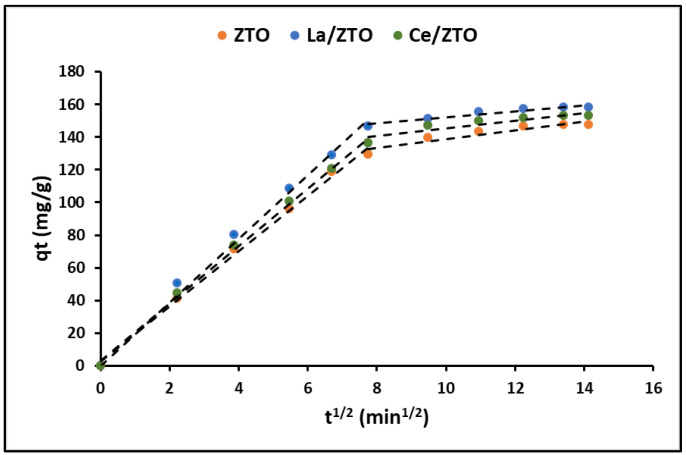
Intraparticle diffusion plots for cyanide removal by nanoparticles.

Since the adsorption rate is determined by the transfer rate of the adsorbate from the solution to the adsorption sites of the adsorbent, the intraparticle diffusion model was also used to describe the nonequilibrium stage of adsorption. Figure 8 shows the variation over time (t^1/2^) of the qt (mg g^−1^) curves for the ZTO, La/ZTO, and Ce/ZTO nanoparticles.

Table 3 shows the cyanide adsorption kinetic parameters calculated for all the nanoparticles.

### 2.4. Photocatalytic Behaviour

Photocatalytic materials (including titanium and zinc oxides) can efficiently degrade organic compounds due to their strong oxidizing capacity, which is generated when these materials are subjected to the action of light. In this study, the photocatalytic activity of ZTO, La/ZTO, and Ce/ZTO nanoparticles was tested by cyanide photodegradation in aqueous solutions, using simulated solar irradiation (λ = 300–800 nm). According to the literature, during the photocatalytic process, the CN^−^ ion can be oxidized to CNO^−^, which in acidic conditions can form HCNO, which is very toxic. However, under basic conditions, the anodic change in the electrical potential of the photogenerated holes (h^+^) as well as the increase in the negative charge on the catalyst surface could decrease its efficiency for cyanide photodegradation [72]. Therefore, in this study, the photocatalytic degradation experiments were performed at pH = 7.

**Figure 9:**
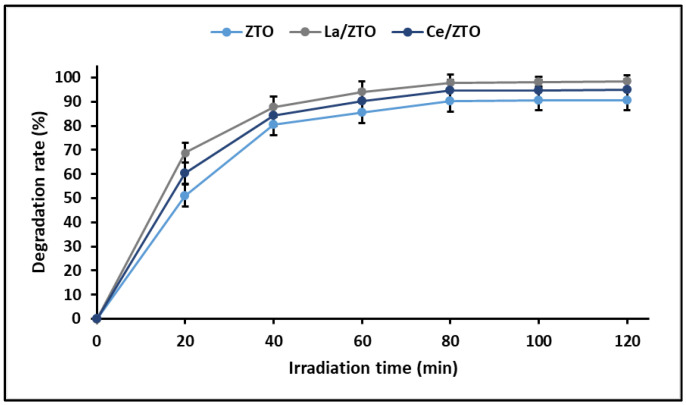
Photocatalytic cyanide degradation by the nanoparticles.

The linear relationship between ln(C_0_/C_t_) and t from the Langmuir–Hinshelwood equation indicated that the photocatalytic degradation reaction also involves the pseudo-first-order reaction, where the apparent rate (k_app_) constants were calculated at 0.022, 0.035, and 0.026 min^−1^ for ZTO, La/ZTO, and Ce/ZTO nanoparticles, respectively. These results are in agreement with those reported by other authors [72]. Figure 9 shows the results obtained in the photocatalytic degradation test. In this figure, it can be seen that the maximum rate of cyanide degradation for the three nanoparticles is reached around the first 90 min, after which the photodegradation becomes constant. The maximum efficiency was achieved by La/ZTO (98.5%), followed by Ce/ZTO (95.1%) and ZTO (90.7%).

Finally, it is widely known that photocatalysis is a reaction that occurs at the solid surface or at the solid–liquid interface, so the photocatalytic efficiency is highly dependent on the specific surface area (SSA) of the catalysts [73]. The results of this study support this argument since it was shown that there is a direct correlation (R^2^ = 0.98) between the specific surface area of nanoparticles and their photocatalytic efficiency. Indeed, the photodegradation of cyanide species was efficient following the order ZTO (90.7%) < Ce/ZTO (95.1%) < La/ZTO (98.5%), which coincides linearly with the order of increase in the SSA of the nanoparticles, that is, ZTO (93 m^2^ g^−1^) < Ce/ZTO (112 m^2^ g^−1^) < La/ZTO (128 m^2^ g^−1^). This probably occurs because a larger SSA provides a greater number of active sites available for reacting with adsorbed water molecules and photogenerated ROS, as well as for the adsorption of cyanide species that will subsequently be photodegraded.

### 2.5. Reuse of the Nanoparticles

**Figure 10:**
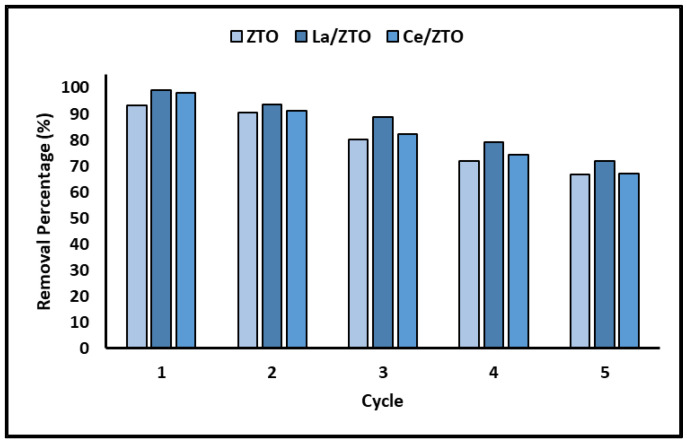
Percentage of cyanide removal for five successive cycles.

Since the stability and recyclability of the materials with adsorbent and photocatalytic applications are considered important factors for their large-scale application, several consecutive cycles of cyanide removal were performed for the ZTO, La/ZTO, and Ce/ZTO nanoparticles. Figure 10 shows the removal efficiency of these nanoparticles during five consecutive cycles.

## 3. Discussion

### 3.1. Characterization of the Nanoparticles

#### 3.1.1. XRD and SSA Analysis

Figure 1 shows that the doping of La and Ce ions decreases the intensity of the ZnTiO_3_ peaks, demonstrating that both dopants have a significant influence on its crystalline structure, which will definitely influence the adsorbent and photocatalytic activity of this semiconductor [74]. It is widely known that the ionic radii of La^3+^ (0.113 nm), Ce^3+^ (0.111 nm), and Ce^4+^ (0.101 nm) are much larger than those of Ti^4+^ (0.068 nm), so it is very difficult for these lanthanide ions to replace the Ti^4+^ in the ZnTiO_3_ crystal lattice. In fact, several studies in the literature have reported that La and Ce ions do not enter the crystalline structure of titanium oxides, but are evenly dispersed on the surface in the form of La_2_O_3_, Ce_2_O_3_/CeO_2_ particles [56]. Although Figure 1 does not show characteristic peaks of these oxides, probably due to their low concentration (1%) and good superficial dispersion [39], the decrease in the size of the crystallites would be a consequence of the presence of Ce-O-Ti or La-O-Ti on the surface of ZnTiO_3_. Indeed, the reduction in the size of the crystallites would result from the influence of the contraction by Re-O-Ti bonds, as several authors have reported [44]. The results of the BET analysis would corroborate this appreciation since the doped nanoparticles show a higher specific surface area for La/ZTO and Ce/ZTO compared to ZTO. From the results of the BET analysis, it is suggested that the higher surface area of La/ZTO and Ce/ZTO compared to ZTO could lead to a higher removal of cyanide species due to the availability of a much more active surface. Furthermore, the high surface area enhances the diffusion of cyanide species and photogenerated active radicals, which promotes cyanide photodegradation by allowing more energetic photons to be adsorbed on the surface of the photocatalyst nanoparticles. Finally, the high surface area facilitates the contact between the cyanide and the respective nanoparticles, which benefits the subsequent photocatalytic reaction [72].

#### 3.1.2. SEM and EDS Analysis

The effect of the La and Ce dopants on the morphology of ZnTiO_3_ was also evidenced by contrasting the SEM images of ZTO, La/ZTO, and Ce/ZTO in Figure 2. As shown in this figure, the La/ZTO and Ce/ZTO nanoparticles are smaller than ZTO nanoparticles. According to the literature, this is because the dispersion of the La^3+^ and Ce^3+/4+^ doping ions on the surface of the ZTO crystallites restricts direct contact with neighboring crystallites, which inhibits their growth and stabilizes the smaller particles [74]. These changes are in agreement with the results deduced from the XRD patterns that confirm the incorporation of heterogeneous elements on the semiconductor surface. Indeed, the incorporation of La and Ce into the ZTO nanoparticles was confirmed by energy-dispersive X-ray spectroscopy (EDS) (Figure 2). According to this analysis, the La and Ce dopants were incorporated into ZTO at a percentage of approximately 1.19%.

#### 3.1.3. Optical and Photoelectric Properties

In this study, the absorption threshold of the undoped ZnTiO_3_ semiconductor was approximately 400 nm. Figure 3a shows that the ZnTiO_3_ semiconductor exhibits a strong absorption band in the border region between UV and visible light. This absorption band is due to the electronic transition that occurs between the valence band (VB) and the conduction band (CB) of the semiconductor. According to a previous study, the VB of ZnTiO_3_ is mainly dominated by the 3d and 2p orbitals of Zn and O, respectively, while the CB of this semiconductor is mainly dominated by the 3d orbital of Ti [64]. Figure 3a further shows that the adsorption threshold of La/ZTO and Ce/ZTO is shifted slightly towards the visible light region, which means that doping with La and Ce ions could enhance the response of ZTO to visible light. However, Figure 3b reveals that the bandgap energies (E_g_) of La/ZTO and Ce/ZTO are less than the bandgap energy of ZTO. As is known, the band gap determines the recombination rate of the electron/hole pairs (e^−^/h^+^), so the decrease in the band gap energy between the occupied and unoccupied bands in the doped nanoparticles would have a fundamental role in the photocatalytic activity of these semiconductors under solar radiation [11].

### 3.2. Effect of Nanoparticles’ Composition

In this study, a preliminary evaluation of the removal of cyanide species was developed to estimate the proper loading of the doping elements (La and Ce) in the ZTO nanoparticles. Figure 4 shows that increasing the proportion of dopants in the ZTO nanoparticles from 0.5 to 1 wt% led to better cyanide species removal capacity. However, by increasing the load of doping elements from 1 to 2 wt%, a decrease in the removal capacity of said species was observed. This agrees with what has been reported by several authors, who have demonstrated the efficacy of the use of lanthanides La and Ce ≤ 1–2 wt% to dope semiconductors and improve their photocatalytic activity in a wide range of the electromagnetic spectrum [39]. In fact, the literature indicates that the use of high concentrations of these dopants is counterproductive, since it can increase the number of oxygen vacancies and, therefore, increase the recombination centers of photoinduced pairs (e^−^/h^+^). Furthermore, due to a high surface agglomeration of dopant particles, the active sites on the catalyst surface could be blocked, thus decreasing its photocatalytic efficiency [47,75].

### 3.3. Adsorption Behaviour

#### 3.3.1. Effect of the pH of the Solution

It is widely known that the surface charge of metal oxides depends on the pH of the solution since it is influenced by the degree of protonation/deprotonation of the surface hydroxyl groups. Consequently, these oxides are positively charged under acidic conditions and negatively charged under basic conditions [16]. Between the two pH regions, acidic and basic, lies the point of zero charge (PZC), that is, the pH at which the total charge on the surface of the material is zero. It is important to note that the PZC of a material is not fixed but could be displaced by the presence of dopant ions on its surface. This is because dopant ions can alter the charge density and electrical potential of the material surface [76,77].

Some materials, such as titanium oxide (TiO_2_), which are considered weak Brönsted solid acids, can easily hydrate and form surface hydroxyl groups (Ti-OH) [14]. These OH groups are sensitive to changes in pH and can undergo protonation/deprotonation reactions, which generates a pH-dependent surface charge, thus:

(4) $$\mathrm{TiOH}+{\;H}^{+}\;↔{\;\mathrm{TiOH}}_{2}^{+}\;\mathrm{at}\;\mathrm{pH}\;<\;{\mathrm{pH}}_{\mathrm{PZC}}$$

and

(5) $$\mathrm{TiOH}\;↔{\;\mathrm{TiO}}^{-}+H^{+}+H_{2}O\;\mathrm{at}\;\mathrm{pH}\;>\;{\mathrm{pH}}_{\mathrm{PZC}}$$

where TiOH_2_^+^, TiOH, and TiO^−^ are positive, neutral, and negative surface hydroxyl groups, respectively. Furthermore, it has been reported that there is a high probability of the appearance of cationic soluble titanium complexes in acidic solutions, mainly in the form of TiOH_2_^+^ [77].

Figure 5 of this article shows that the amount of cyanide adsorbed increases slightly with increasing pH, reaching a maximum peak at pH = 8. Above this value, cyanide adsorption remains constant in doped nanoparticles (La/ZTO and Ce/ZTO), while for the undoped nanoparticles (ZTO) a slight decrease in adsorption is observed. This is probably because doping with lanthanum (La) and cerium (Ce) leads to a greater total number of available active sites on the outer surface of the doped nanoparticles, resulting in more favorable kinetics [78].

The nanoparticles used in this study showed an average point of zero charge (pH_PZC_) of 7.4, favoring a gradual increase in positively charged clusters towards lower pH values and a gradual increase in negatively charged clusters towards higher pH values. However, it is well known that the degree of dissociation of HCN is small (pH < pKa = 9.4), so at pH > pKa, cyanide in solution dissociates mainly in its ionic form (CN^−^ + H^+^) while at pH < pKa it associates in molecular form in HCN. As can be seen, not only the surface charge of the adsorbents can be affected by the pH of the solution, but also the level of dissociation and speciation of the adsorbate.

Regarding the adsorption of cyanide species on hydrated oxides, several authors have reported that, at pH < pH_PZC_, the HCN species can adsorb on the surface OH groups of the oxide by forming H-N polar covalent bonds, as illustrated below [13].

(6) TiOH+ HCN ↔ TiOH⋯NCH

or

(7) $${\mathrm{TiOH}}_{2}^{+}+\;\mathrm{HCN}\;↔{\;\mathrm{TiOH}}_{2}^{+}\cdots \mathrm{NCH}$$

For pH values < 7.0, the surface of the nanoparticles is positively charged (pH < pH_PZC_ = 7.4), while the degree of dissociation of HCN is small (pH < pKa = 9.4). Therefore, at pH values below pH_PZC_ = 7.4, there is little likelihood of electrostatic forces developing between a charged surface and a neutral molecule, especially at lower pH values. As pH values increase above pH_PZC_ = 7.4, the negative charge on the nanoparticles surface increases, but the dissociation of HCN into CN^−^ ions is still not important; however, an increase in attractive electrostatic forces is expected and therefore an increase in total cyanide adsorption, as concluded from our experimental results. At pH values > pKa = 9.4, the percentage of CN^−^ in the solution increases, but, in parallel, the surface of the nanoparticles is negative since pH > pH_PZC_. Due to this increase in surface negative charge, the cyanide anion can hardly be adsorbed by the nanoparticles at very basic pH, since repulsive forces are generated between the adsorbent and the adsorbate.

The low adsorption of cyanide species on ZTO nanoparticles at high and low pH values, as shown in Figure 5, suggests that the adsorption process on these nanoparticles is mainly governed by electrostatic interactions, although the participation of other types of interactions cannot be totally excluded (Van der Waals and/or specific). Indeed, CN^−^ adsorption on La/ZTO and Ce/ZTO doped nanoparticles could occur predominantly by chemical adsorption rather than physical adsorption (outer sphere complex), although physical absorption may also be present. Our results were in accordance with those reported in the literature [79]. When CN^−^ is in contact with active cationic groups on the adsorbent surface, it forms a bond with them. According to the literature, both lanthanum and cerium show a strong electron withdrawal effect [55,80], so the presence of these rare earth elements on the surface of the ZTO nanoparticles could contribute to the formation of Lewis acid sites [52,81,82], which would improve cyanide adsorption on its surface [78], in addition to providing catalytic stability in an aqueous reaction medium [83].

#### 3.3.2. Adsorption Isotherm

The adsorption isotherms shown in Figure 6 show that the adsorption rate is enhanced at low concentrations of cyanide species (HCN + CN^−^); however, at higher concentrations, there are more cyanide species competing for the available active sites on the surface of the adsorbent nanoparticles, so the rate of adsorption reaches a maximum (saturation) where it stabilizes. Consequently, the initial concentration of the cyanide species in the solution generates an important driving force to overcome the resistance to mass transfer of these species from the liquid phase to the surface of the nanoparticles [12].

Experimental equilibrium adsorption data for the three materials (ZTO, La/ZTO, and Ce/ZTO) were fitted to the Langmuir and Freundlich isotherm models. The parameters corresponding to the adjustment of these experimental results are summarized in Table 2. As shown in the table, the highest values of R^2^ indicted that, for all three materials, the Langmuir model was more suitable to fit the equilibrium adsorption data. Therefore, it is suggested that the adsorption of cyanide species on these adsorbents occurs as a phenomenon of electrostatic attraction and corresponds to a monolayer adsorption on sites with homogeneous surfaces [12]. In addition, the favorability of cyanide adsorption on nanoparticles could be confirmed from the values of the Langmuir and Freundlich constants (R_L_ and n), which turned out to be on average 0.14 and 2.7, respectively. This demonstrates that the strongest binding sites are occupied first and that the binding strength decreases with increasing degrees of site occupancy [79].

#### 3.3.3. Adsorption Kinetics

Figure 7 shows the kinetic data of the adsorption of cyanide species obtained experimentally for the nanoparticles (ZTO, La/ZTO, and Ce/ZTO). These data were fitted to pseudo-first-order (PFO) and pseudo-second-order (PSO) kinetic models. From this figure, it can be seen that, for all three compounds, the adsorption of cyanide species was rapid in the first 60 min, after which the adsorption remained constant. This behavior is due to the fact that, initially, the three compounds had available adsorption sites, which were progressively occupied until they prevented the adsorption of more cyanide species. Table 4 shows the kinetic parameters of adsorption obtained in the present study. As can be seen in the table, there was a better fit of the experimental data (higher correlation coefficient (R^2^)) to the PSO model, which suggests a process of chemisorption of cyanide species on the surface of the nanoparticles [84].

The fit of the experimental data to the intraparticle diffusion model is shown in Figure 8. This figure shows that the cyanide adsorption process on nanoparticles is conceptually divided into two steps (linear regions), after which intrinsic adsorption could occur by physical and chemical adsorption or binding to an appropriate active site of the nanoparticles. The initial fast-rate stage could be described as a process of cyanide diffusion through the stationary film surrounding each adsorbent and the transfer of the bulk solution to the adsorbent surface. In contrast, the low-velocity second stage suggests a diffusion process through the pores, corresponding to intraparticle mass transfer. Table 4 also summarizes the linear regression analysis for the diffusion kinetic models. Given the relatively high values of A found in this study, it is suggested that surface adsorption could be the rate-limiting step [79].

### 3.4. Photocatalytic Behaviour

Regarding the photocatalytic behavior of the nanoparticles, in this study, it was possible to show that the nanoparticles ZTO, La/ZTO, and Ce/ZTO achieved an effective removal of cyanide in an aqueous solution. Apparently, doping with lanthanum and cerium allowed, on the one hand, an increase in the number of more active centers for the adequate adsorption of the target molecule in a specific surface area that was improved with respect to ZTO. On the other hand, the presence of La and Ce on the surface of ZTO allowed for a decrease in the energy of the bandgap, which could contribute to improving the photocatalytic activity of the nanoparticles. In fact, the bandgap plays a fundamental role in the photocatalytic activity of semiconductors since it participates in determining the recombination rate of electron/hole pairs (e^−^/h^+^) [11]. In this study, it was shown that La/ZTO and Ce/ZTO had lower bandgap energy than ZTO, which suggests that, given the smaller separation between the valence and conduction bands, both doped nanoparticles could easily transfer photoinduced electrons from the bulk to the surface and be more active than the ZTO nanoparticles under simulated solar radiation [85].

**Table 4:** Comparison of the adsorption capacity (mg g^−1^) of various materials for cyanide removal.

Adsorbent	q_max_ (mg g^−1^)	Isotherm Model	Kinetic Model	Reference
Clay-K	253.98	-	Pseudo-second-order	[12]
TiO_2_/Fe_2_O_3_	124.87	-	Pseudo-second-order	[12]
LTA zeolite modified with HDMTMAB	24.09	Langmuir	-	[79]
ZnO	275	Langmuir	Pseudo-second-order	[84]
NiO	185	Langmuir	Pseudo-first-order	[84]
ZnO-NiO	320	Langmuir	Pseudo-second-order	[84]
Fe-MFI zeolite	33.98	Langmuir	Pseudo-second-order	[86]
ZnTiO_3_	57.32	Langmuir	Pseudo-second-order	In this study
La/ZnTiO_3_	59.22	Langmuir	Pseudo-second-order	In this study
Ce/ZnTiO_3_	42.00	Langmuir	Pseudo-second-order	In this study

The results of this study allow us to suggest that the existence of La and Ce ions on the surface of ZTO could influence the photoactivity of this photocatalyst by altering the recombination rate of the pair (e^−^/h^+^). Both La/ZTO and Ce/ZTO could initiate the photocatalytic process in which they participate, with the excitation of electrons (e^−^) under simulated sunlight. These excited electrons would be immediately transferred from the valence band (VB) to the conduction band (CB) of the photocatalyst, leaving a hole (h^+^) in the VB (reaction R1) due to the formation of a pair (e^−^/h^+^). The (e^−^/h^+^) pairs could migrate to the photocatalyst surface where they would react directly with adsorbed species, such as H_2_O, OH^−^, O_2_, and other (R) molecules, including cyanide. However, the (e^−^/h^+^) pairs could also recombine immediately upon formation (Equation 9). The (h^+^) generated in the VB of the photocatalyst could oxidize both adsorbed water molecules and hydroxyl ions in order to generate highly reactive hydroxyl radicals (Equations 10 and 11). The migration of the holes (h^+^) towards the surface of the photocatalyst would allow for prolonging their useful life and at the same time create more reactive radicals to oxidize the molecules adsorbed on said surface. Both the La ion and the Ce ion have completely empty 5d orbitals that could confine photoexcited (e^−^) in the CB of the photocatalyst (Equation 12); however, these (e^−^) are highly unstable, so they can migrate to the surface of the photocatalyst where they react with adsorbed oxygenated molecules, to generate O_2_^−^ and OH˙ radicals through a sequence of reactions (Equations 13–16). These radicals have sufficient reducing potential to readily oxidize organic compounds, including cyanide (Equation 17) [85]. Finally, the direct oxidation of cyanide is also possible as long as the molecule reacts directly with (h^+^) photogenerated (Equation 18) [44]. The following reaction scheme suggests a likely route for cyanide photodegradation on the surface of M^(n+)/^ZTO (M^(n+)^ = La^(3+)^ or Ce^(3+/4+)^) [81]:

(8) $$M^{n+}/\mathrm{ZTO}\overset{\mathrm{hv}}{\to }{\;M}^{n+}/\mathrm{ZTO}\;+{\;e}_{\mathrm{CB}}^{-}+{\;h}_{\mathrm{VB}}^{+}$$

(9) $$e_{\mathrm{CB}}^{-}+{\;h}_{\mathrm{VB}}^{+}\to \mathrm{heat}$$

(10) $$H_{2}O_{\mathrm{ads}}+{\;h}_{\mathrm{VB}}^{+}\;⇌\;{\left(H^{+}+{\;\mathrm{OH}}^{-}\right)}_{\mathrm{ads}}+{\;h}_{\mathrm{VB}}^{+}\to {\mathrm{OH}}_{\mathrm{ads}}^{⦁}$$

(11) $${\mathrm{OH}}_{\mathrm{ads}}^{-}+{\;h}_{\mathrm{VB}}^{+}\to {\;\mathrm{OH}}_{\mathrm{ads}}^{⦁}$$

(12) $$M^{\left(n+\right)}+e_{\mathrm{CB}}^{-}\;\to {\;M}^{\left(n+\right)-1}$$

(13) $$M^{\left(n+\right)-1}+{\left(O_{2}\right)}_{\mathrm{ads}}\;\to {\;M}^{\left(n+\right)}+{\;O}_{2}^{⦁-}$$

(14) $$O_{2}^{⦁-}+{\;H}^{+}\to {\;\mathrm{HO}}_{2}^{⦁}$$

(15) $$2{\mathrm{HO}}_{2}^{⦁}\to {\;H}_{2}O_{2}+{\;O}_{2}$$

(16) $$H_{2}O_{2}+{\;e}_{\mathrm{BC}}^{-}\to {\;\mathrm{OH}}^{⦁}+{\;\mathrm{OH}}^{-}$$

(17) $$R+{\;\mathrm{OH}}_{\mathrm{ads}}^{⦁}\to {\;R}_{\mathrm{ads}}^{\prime ⦁}+{\;H}_{2}O\to \mathrm{degradation}\;\mathrm{products}$$

(18) $$R_{\mathrm{ads}}+{\;h}_{\mathrm{VB}}^{+}\to {\;R}_{\mathrm{ads}}^{⦁+}\to \mathrm{degradation}\;\mathrm{products}$$

### 3.5. Reuse of the Nanoparticles

Chemical stability is a particularly important property, which is directly related to the useful life of the material and its possible applications. Figure 10 clearly demonstrates that the percentage of cyanide removal decreases with increasing cycle times. However, after five consecutive treatment cycles, the loss of activity of the evaluated materials did not exceed 30% on average. Consequently, ZTO, La/ZTO, and Ce/ZTO still have good activity and can efficiently remove cyanide species in an aqueous solution.

**Figure 11:**
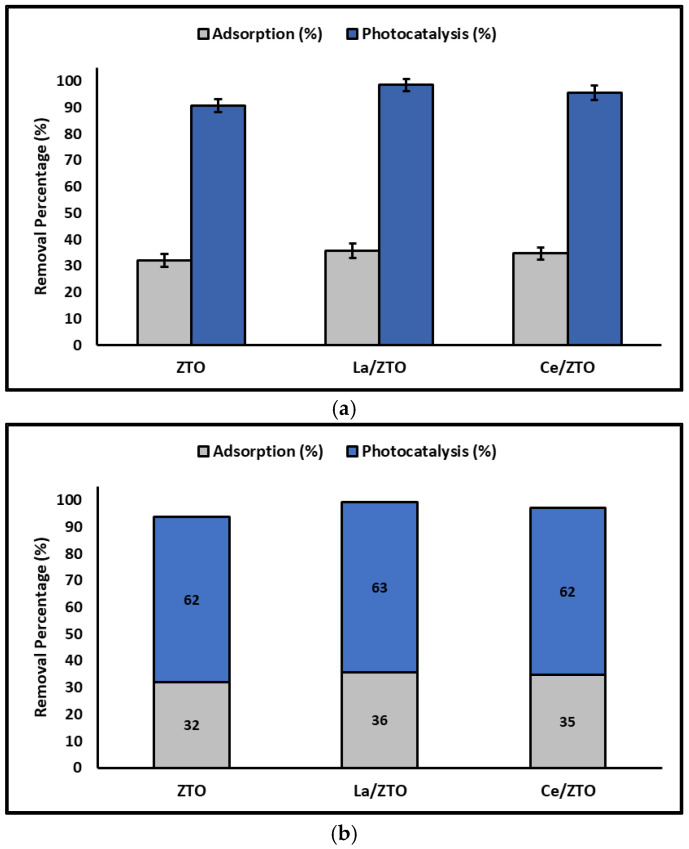
Percentage of (**a**) individual and (**b**) accumulated cyanide adsorbed and photodegraded by the nanoparticles.

Figure 11 shows the individual and accumulated percentages of cyanide adsorbed and photodegraded by the nanoparticles synthesized in this study.

Finally, Table 4 compares the maximum adsorption capacity (mg g^−1^) of the present nanoparticles and some adsorbents used for cyanide removal from aqueous solutions.

Likewise, Table 5 compares the photodegradation efficiency (%) of the present nanoparticles and some photocatalysts used for cyanide removal from aqueous solutions.

Considering and comparing the adsorption and photodegradation results obtained in this study with those reported in the literature cited above, it is suggested that La/ZTO, Ce/ZTO, and ZTO nanoparticles, in that order, are effective for the removal of species of cyanide from aqueous systems. Furthermore, it is suggested that the removal of cyanide species was governed by a combination of electrostatic interactions and formation of complexes by coordinate covalent bonds, followed by photo-oxidation from the surface of the oxides when the reaction systems were subjected to simulated solar light.

## 4. Materials and Methods

### 4.1. Materials

The following reagents (analytical grade) were used in this study without further purification: isopropyl alcohol (C_3_H_8_O, Sigma Aldrich, St. Louis, MO, USA, ≥99.5%), titanium (IV) isopropoxide (Ti(OC_3_H_7_)_4_, Sigma Aldrich, St. Louis, MO, USA, 98.0%), acetic acid (CH_3_COOH, Sigma Aldrich, St. Louis, MO, USA, 99.8%), zinc acetate dihydrate (Zn(CH_3_COO)_2_∙2H_2_O, ACS, St. Louis, MO, USA, ≥98.0%), lanthanum nitrate hexahydrate (La(NO_3_)_3_∙6H_2_O, Sigma Aldrich, St. Louis, MO, USA, 99.9%), cerium(III) nitrate hexahydrate (Ce(NO_3_)_3_∙6H_2_O, Sigma Aldrich, St. Louis, MO, USA, 99.9%), potassium cyanide KCN, Sigma Aldrich, St. Louis, MO, USA, ≥97.0%), sodium hydroxide (NaOH, Sigma Aldrich, St. Louis, MO, USA, ≥85.0%), picric acid ((O_2_N)_3_C_6_H_2_OH, Sigma Aldrich, St. Louis, MO, USA, ≥99.0%), sodium carbonate (Na_2_CO_3_, Sigma Aldrich, St. Louis, MO, USA, ≥99.0%).

### 4.2. Synthesis of the Nanoparticles

The ZnTiO_3_ (ZTO), La/ZnTiO_3_ (La/ZTO), and Ce/ZnTiO_3_ (Ce/ZTO) nanoparticles were synthesized following a modified sol-gel method described in previous studies [64]. To obtain the ZTO nanoparticles, an aqueous solution (Solution A) consisting of zinc acetate dihydrate (Zn(acet)), water, and isopropyl alcohol (iPrOH) was prepared at room temperature, using a 1:1 TiO_2_/ZnO molar ratio. The iPrOH/water (50 *v*/*v*%) ratio was determined by stoichiometry, being the amount necessary to hydrolyze titanium (IV) isopropoxide (TIPP) dissolved in isopropyl alcohol (iPrOH) at a TIPP/iPrOH ratio of 70 *v*/*v*% (Solution B). Solution B was slowly added dropwise to solution A maintaining constant stirring and room temperature. After the formation of a white precipitate, the reaction system was stirred for 60 min at room temperature. The precipitate was dried at 60 °C for 24 h and then calcined at 500 °C for 4 h. Finally, the obtained solids were cooled to room temperature. To obtain the La/ZTO and Ce/ZTO doped nanoparticles, the procedure described above was repeated, adding the lanthanum or cerium salts to the aqueous zinc solution to obtain a final concentration of the doping element of ~1% per gram of ZTO.

### 4.3. Characterization of the Nanoparticles

For X-ray diffraction (XRD) measurements, a Bruker-AXS D8-Discover diffractometer (Bruker AXS, Karlsruhe, Germany) was used. Data were recorded from 5 to 70° in the 2θ range, using Cu Kα (1.5406 Å) as a radiation source. The specific surface area (SSA) was determined by the adsorption of liquid nitrogen (−196 °C) from a gas mixture of nitrogen and helium (30% nitrogen). For this, the ChemiSorb 2720 equipment (Micromeritics, Norcross, GA, USA) was used. Micrographs of the synthesized samples were obtained by field effect scanning electron microscopy (SEM) on a Zeiss Gemini ULTRA plus electron microscope (Carl Zeiss AG, Ober-kochen, Germany) operating at 3.0 kV. For SEM measurements, samples were dropped and dried on a silicon wafer. A JEOL JSM 6400 scanning electron microscope (JEOL, Peabody, MA, USA) was used to obtain energy-dispersive X-ray (EDX) spectra. Diffuse UV-Vis reflectance spectra (DRS) were obtained using a Nicolet Evolution 201/220 Thermo UV-Vis spectrophotometer (ThermoFisher, Waltham, MA, USA), fitted with an integrating sphere unit, which uses barium sulfate as a reference. To simulate solar light and evaluate the photoactivity of the nanoparticles, a solar box ATLAS, SUNTEST CPS+ was used, equipped with a 1500 W xenon lamp and air-cooled (Atlas Material Testing Technology, Mount Prospect, IL, USA). In this box, the irradiance was set at 250 Wm^−2^, and no cut-off filter was used, so wavelengths from 300 to 800 nm were allowed to pass. Finally, the amount of cyanide remaining in the solutions was quantified using a Jenway 7350 spectrophotometer (Cole-Parmer, Staffordshire, UK). In XRD patterns, full width at half peak height (FWHM) was determined using MDI JADE, (version 6; data analysis software; Materials Data Inc., Livermore, CA, USA, 2014). The respective crystalline phases were identified using the ICDD database (International Center for Diffraction Data, version 2018). The Chemisoft TPx system (version 1.03; data analysis software; Micromeritics, Norcross, GA, USA, 2011) was used to calculate the SSA by the single-point method using the Brunauer–Emmet–Teller (BET) equation. Finally, IBM SPSS (version 25.0; statistic software for Windows; IBM Corp.; Armonk, NY, USA, 2017) was used for the ANOVA analysis.

### 4.4. Adsorption Behaviour

The alkaline picrate method [92,93] was used with some modifications to quantify total cyanide. To prepare the alkaline picrate solution, 1 g of picric acid and 5 g of sodium carbonate were dissolved in 200 mL of HPLC water. The cyanide calibration curve (R^2^ = 0.9996) was prepared using several cyanide standard solutions (0–40 mg L^−1^) that were obtained from a stock solution (1000 ± 5 mg L^− 1^ KCN in 0.1% NaOH). The total cyanide concentration was determined by adding 4 mL of alkaline picric acid solution to 1 mL of cyanide standard solution or blank (HPLC water) contained in a test tube. This mixture was incubated in a water bath at 95 °C for 5 min, after which the absorbance at 490 nm was measured using a UV-vis spectrophotometer. Assay results were expressed as the average of three replicates [94].

The total cyanide adsorption experiments were performed at room temperature, using a batch method and maintaining the pH of the solutions at 7.0 ± 0.1 by adding 0.1 M HCl or NaOH solutions. To determine the concentration of total cyanide remaining in the solutions, aliquots of the reaction system were withdrawn using a syringe. Aliquots withdrawn at defined time intervals were filtered through a 0.45 µm membrane to remove any suspended particles that might interfere with the measurement. The total cyanide concentration remaining in the solution was determined using the alkaline picrate method described above. All tests were performed in triplicate. This procedure was repeated without adding the adsorbent nanoparticles to the cyanide solution, in order to eliminate any photolysis effect due to natural light. The amount of cyanide adsorbed was calculated using the following expression [65]:

(19) $$q_{e}=\left(C_{0}-{\;C}_{e}\right)\times \frac{v}{w}$$

where C_0_ and C_e_ are expressed in mg L^−1^ and correspond to the initial and equilibrium concentration, respectively; v is the volume of the solution expressed in liters (L); and w is the mass of the adsorbent expressed in grams (g).

#### 4.4.1. Effect of the pH of the Solution

The effect of pH on the adsorption of cyanide species was investigated for all nanoparticles using solutions with different pH values, from 3 to 12. For all experiments, the initial cyanide concentration was 20 mg mL^−1^ and the time of contact was 180 min. To evaluate the effect of pH on the cyanide removal capacity of nanoparticles, their point of zero charge (PZC) at room temperature (20 ± 2 °C) was also determined using the pH drift method (ΔpH = pH_f_ − pH_i_ = 0). The assay was carried out in a series of 50 mL centrifuge tubes containing 25 mL of a 0.1 M NaCl solution to which 0.1 g of solid sample was added. The pH of the solutions contained in each tube was adjusted with 0.1 M HCl and 0.1 M NaOH solutions to obtain the desired pH value in the range of 3–12. The pH of the supernatant liquid in each tube was called pH_i_. The tubes containing the samples were stirred at 220 rpm for 24 h. After this time, the pH of the supernatant liquid in each tube was measured again and it was called pH_f_. The PZC was obtained from the graph of ΔpH (ΔpH = pH_f_ − pH_i_) vs. pH_i_. Assays were replicated using 0.01 and 0.05 M NaCl solutions. All experiments were performed in triplicate and the average value was reported [95].

#### 4.4.2. Adsorption Isotherm

The effect of the initial cyanide concentration on the adsorption capacity of the nanoparticles was investigated by varying the cyanide concentration from 0.20 to 40 mg L^−1^. The experiments were carried out at room temperature and neutral pH. Equilibrium total cyanide adsorption was evaluated according to the well-known Langmuir and Freundlich isotherm models. The Langmuir isotherm model can be represented by the following expression [84]:

(20) $$\frac{C_{e}}{q_{e}}=\frac{1}{K_{L}q_{\max}}+\frac{C_{e}}{q_{\max}}$$

where q_max_ is expressed in mg g^−1^ and represents the maximum monolayer adsorption; K_L_ is expressed in L mg^−1^ and represents the equilibrium Langmuir constant related to the adsorption energy; and C_e_ is expressed in mg L^−1^ and represents the concentration of solute at equilibrium. Additionally, the R_L_ separation factor values, which provide an insight into the adsorption nature, can be expressed using the following expression [84]:

(21) $$R_{L}=\frac{1}{\left(1+K_{L}C_{e}\right)}$$

The Freundlich isotherm model can be represented by the following expression [84]:

(22) qe= KFCe1n

where K_F_ is expressed in L mg^−1^ and represents the Freundlich constant, which indicates the adsorption affinity of the adsorbents, and 1/n is another constant that represents the adsorption intensity. For favorable adsorption, the value of the constant n should be in the range of 1 to 10 [79].

#### 4.4.3. Adsorption Kinetics

To evaluate the effect of contact time on the total cyanide adsorption capacity of nanoparticles, batch kinetics experiments were performed under similar conditions to those mentioned above for equilibration experiments. Typically, 20 mg of the nanoparticles were stirred magnetically in an aqueous cyanide solution (100 mL of water containing 20 mg L^−1^ potassium cyanide).

The total cyanide concentration was determined using aliquots that were withdrawn from the reaction system at regular time intervals. In this study, the kinetics of solute absorption at the solute–solution interface was determined using reaction-based models (pseudo-first-order and pseudo-second-order), as well as models based on transport phenomena (intraparticle diffusion, film diffusion, external and internal pore diffusion). The pseudo-first-order kinetic model is represented by the following expression [79]:

(23) $$\ln\left(q_{e}-q_{t}\right)=\ln\left(q_{e}\right)-{\;k}_{1}t$$

where q_e_ and q_t_ are expressed in mg g^−1^ and represent the cyanide adsorbed per unit weight at equilibrium and at any time t, respectively, and k_1_ is expressed in min^−1^ and represents the rate constant.

The pseudo-second-order kinetic is represented by the following expression [79]:

(24) $$\frac{t}{q_{t}}=\frac{1}{k_{2}q_{e}^{2}}+\frac{1}{q_{e}}t$$

where k_2_ is expressed in g mg^−1^ min^−1^ and represents the pseudo-second-order rate constant.

To get a good understanding of the cyanide adsorption mechanism on the nanoparticles’ surface, the rate-limiting step in the adsorption process was also determined. The intraparticle diffusion model assumes that intraparticle diffusion is generally the rate-controlling step in well-mixed solutions. The intraparticle diffusion model is described by the following expression [79]:

(25) qt= k3t12+A

where k_3_ is expressed in mg g^−1^ min^−1/2^ and represents the intraparticle diffusion rate constant and A is expressed in mg g^−1^ and represents a constant indicating the thickness of the boundary layer, such that, the greater the value of A, the greater the effect of the boundary layer. When the q_t_ plot against the square root of time displays multilinearity, it means that the diffusion occurs in several steps during the process.

Finally, the data obtained from the adsorption kinetics experiment were fitted to the internal pore diffusion model. Therefore, when particle diffusion controls the process, the adsorption rate is described by the following expression [79]:

(26) $$-\ln\left(1-{\left(\frac{q_{t}}{q_{e}}\right)}^{2}\right)=\frac{2\pi^{2}D_{p}}{r^{2}}\;t$$

However, when the external film diffusion controls the process, the adsorption rate is described by the following expression [79]:

(27) $$-\ln\left(1-\left(\frac{q_{t}}{q_{e}}\right)\right)=\frac{D_{f}C_{s}}{{h\;r\;C}_{z}}\;t$$

where q_t_ and q_e_ are expressed in mg g^−1^ and represent the solute loads in the adsorbent phase at time t and at equilibrium, respectively; t is the contact time expressed in minutes (min); C_s_ (mg L^−1^) and C_z_ (mg kg^−1^) are the ion concentrations in the solution and in the adsorbent, respectively; r is the average radius of the adsorbent nanoparticles (1 × 10^−7^ m); and h is the thickness of the film that surrounds the nanoparticles, which is considered as h = 10^−6^ m for slightly stirred solutions [96]. D_p_ and D_f_ are expressed in m^2^ min^−1^ and correspond to the diffusion coefficient in the adsorbent phase and the diffusion coefficient in the film phase surrounding the adsorbent particles, respectively.

### 4.5. Photocatalytic Behaviour

The heterogeneous photocatalysis experiments were performed at room temperature keeping the pH of the solution at 7.0 ± 0.1. Typically, 20 mg of the nanoparticles was stirred magnetically in an aqueous cyanide solution (100 mL of water containing 20 mg L^−1^ potassium cyanide). The solution was kept under dark conditions for 30 min to reach adsorption–desorption equilibrium. Then the photoactivity of the nanoparticles was evaluated under simulated sunlight radiation. The remaining total cyanide concentrations were determined by UV-Vis spectrophotometry at 490 nm by the picrate method. Samples were withdrawn at 10 min intervals with a syringe and filtered through a 0.45 µm membrane filter to remove any solid particles interfering with the measurement. All tests were performed in triplicate using a potassium cyanide solution without nanoparticles irradiated with simulated sunlight as a blank to eliminate any photolysis effect.

The photocatalytic degradation rate of cyanide species in the heterogeneous photocatalytic systems under simulated sunlight for the ZTO, La/ZTO, and Ce/ZTO nanoparticles was followed by the Langmuir–Hinshelwood equation [97], which can be expressed by the following expression [72]:

(28) $$\ln\frac{C_{o}}{C_{t}}=\mathrm{kKt}={\;k}_{\mathrm{app}}t$$

where k (min^−1^) is the actual rate constant, K is the adsorption constant of the substrate on the nanoparticles, C_0_ (mg L^−1^) is the initial concentration of the substrate, C_t_ (mg L^−1^) is the concentration at time t (min), and k_app_ (min^−1^) is the apparent rate constant. Plotting ln(C_0_/C_t_) against time t gives the apparent rate constant (k_app_) for substrate degradation from the slope of the curve fit line and the intercept is equal to zero.

### 4.6. Reuse of the Nanoparticles

To determine the reusability of the nanoparticles in cyanide photodegradation, a recycling experiment of several consecutive cycles under the same operating conditions was designed. After completing each photodegradation treatment cycle, the suspensions were left at rest for 60 min to precipitate the nanoparticles. Then, the clear solution was removed from the reaction system and 100 mL of fresh potassium cyanide solution (20 mg L^−1^) was injected into the reaction system, starting the next photocatalytic cycle. The recycling experiment was carried out for five consecutive cycles.

## 5. Conclusions

In summary, from the evidence of this study, it can be concluded that the sol-gel method is suitable for preparing undoped ZnTiO_3_ (ZTO) and doped La (La/ZTO) and Ce (Ce/ZTO) nanoparticles for the effective removal of cyanide species in aqueous solutions.

In general, the experimental data best fit the Langmuir isotherm model and the pseudo-second-order kinetic model. Therefore, in this study, it was evidenced that total cyanide adsorption on ZTO, La/ZTO, and Ce/ZTO nanoparticles occurs as a chemisorption process. In this process, the cyanide species would be deposited forming a single layer on the surface of the nanoparticles, which would be provided with an indefinite number of identical sites. La and Ce ions had a significant effect on the bandgap energy, particle size, and specific surface area of the ZTO semiconductor. These physicochemical changes contributed to improve the adsorbent capacity and photocatalytic activity under simulated sunlight of the semiconductor. In this study, it was found that La/ZTO nanoparticles were the most efficient (99.0%) followed by Ce/ZTO (97.0%) and ZTO (93.6%) for the removal of cyanide species through processes adsorption and photodegradation. Furthermore, it was observed that the synthesized nanoparticles can be recycled up to five times with a total reduction of 30% in the total removal capacity of cyanide species in aqueous solutions. Finally, the results of this study contribute to the potential generation of efficient technologies for the treatment and recovery of our planet’s water resources.

## Figures and Tables

**Table 1:** Effect of nanoparticles’ compositions on their cyanide adsorption capacity.

Nanoparticles’ Composition	HSD Tukey *	Duncan *
	q_e_ (mg g^−1^)	q_e_ (mg g^−1^)
La/ZTO (1.0%)	0.15 ± 0.07 ^a^	0.15 ± 0.07 ^a^
La/ZTO (0.5%)	1.14 ± 0.15 ^b^	1.14 ± 0.15 ^b^
Ce/ZTO (1.0%)	2.46 ± 0.32 ^c^	2.46 ± 0.32 ^c^
La/ZTO (2.0%)	2.78 ± 0.21 ^c,d^	2.78 ± 0.21 ^c^
Ce/ZTO (0.5%)	3.43 ± 0.48 ^d,e^	3.43 ± 0.48 ^d^
Ce/ZTO (2.0%)	3.79 ± 0.36 ^e^	3.79 ± 0.36 ^d^
ZTO	4.93 ± 0.33 ^f^	4.93 ± 0.33 ^e^
*p*-value	<0.001	<0.001

The means for the groups in the homogeneous subsets are displayed. Different lowercase letters (^a–f^) indicate significant differences between groups (*p* < 0.01). * Use the sample size of the harmonic mean = 3.0.

**Table 2:** Isotherm parameters for cyanide sorption on nanoparticles.

Isotherm Parameters		ZTO	La/ZTO	Ce/ZTO
Langmuir	q_max_ (mg g^−1^)	57.32 (±3.53)	59.22 (±2.12)	42.00 (±2.26)
	K_L_ (L mg^−1^)	0.25 (±0.05)	0.38 (±0.04)	0.31 (±0.04)
	R_L_	0.16	0.12	0.14
	χ^2^	6.18	5.03	4.92
	R^2^	0.97	0.99	0.99
Freundlich	K_F_ (L mg^−1^)	14.77 (±2.82)	19.76 (±2.25)	16.72 (±2.55)
	n	2.52 (±0.46)	2.96 (±0.39)	2.67 (±0.43)
	1/n	0.40	0.34	0.37
	χ^2^	4.15	5.96	6.78
	R^2^	0.85	0.93	0.89

**Table 3:** Kinetic parameters for cyanide sorption on nanoparticles.

Kinetic Parameters		ZTO	La/ZTO	Ce/ZTO
Pseudo-first-order	q_max_ (mg g^−1^)	144.73 (±2.81)	151.37 (±2.59)	145.76 (±2.94)
	*k*_1_ (L mg^−1^)	0.04 (±3.35 × 10^−3^)	0.08 (±1.51 × 10^−2^)	0.08 (±1.38 × 10^−2^)
	χ^2^	14.74	15.95	16.24
	R^2^	0.99	0.94	0.93
Pseudo-second-order	q_max_ (mg g^−1^)	163.60 (±2.60)	163.33 (±2.19)	158.07 (±2.44)
	*k*_2_ (L mg^−1^)	3.39 × 10^−4^ (±2.92 × 10^−5^)	7.88 × 10^−4^ (±1.11 × 10^−4^)	7.76 × 10^−4^ (±1.20 × 10^−4^)
	χ^2^	12.31	14.33	13.78
	R^2^	1.00	0.99	0.98
Intraparticle diffusion	*k*_3_ (mg g^−1^ min^−1/2^)	10.01 (±0.24)	10.23 (±0.28)	10.35 (±0.26)
	*A*	28.91 (±1.75)	38.52 (±1.25)	37.65 (±1.57)
	R^2^	0.87	0.84	0.83
External-film diffusion	D*f* (m^2^ min^−1^)	1.16 × 10^−11^	1.38 × 10^−11^	1.38 × 10^−11^
	R^2^	0.99	0.99	0.99
Internal-pore diffusion	D*p* (m^2^ min^−1^)	1.41 × 10^−17^	1.60 × 10^−17^	1.60 × 10^−17^
	R^2^	0.99	0.99	0.99

**Table 5:** Comparison of the photodegradation efficiency (%) of various materials for cyanide removal.

Material	[CN] (mg L^−1^]	[Catalyst] (g L^−1^]	Time (min)	Efficiency (%)	Reference
Cts-Ag	71.6	2.5	180	98.0	[3]
Blast furnace sludge (BFS)	750	2.0	120	97.0	[12]
TiO_2_/Fe_2_O_3_/PAC	300	1.4	170	97.0	[72]
TiO_2_/Fe_2_O_3_/zeolite	200	1.4	160	89.0	[72]
Fe^2+^	10	0.14	30	86.0	[87]
TiO_2_	30	0.05	60	72.0	[88]
Co/TiO_2_/SiO_2_	100	2.0	60	55.0	[89]
TiO_2_/SiO_2_	100	1.7	180	93.0	[90]
Ce/ZnO	250	4.0	180	84.0	[91]
ZnTiO_3_	20.0	0.2	90	90.7	In this study
La/ZnTiO_3_	20.0	0.2	90	98.5	In this study
Ce/ZnTiO_3_	20.0	0.2	90	95.1	In this study

## Data Availability

Data are contained within the article.

## Supplementary Materials

The supporting information can be downloaded at: https://www.mdpi.com/article/10.3390/ijms24043780/s1.
