# Supplementary material for: Comparative Study of the Effect of Doping ZnTiO3 with Rare Earths (La and Ce) on the Adsorption and Photodegradation of Cyanide in Aqueous Systems
Source: Int J Mol Sci. 2023 Feb 14;24(4):3780. doi: 10.3390/ijms24043780 (PMC9960395; doi:10.3390/ijms24043780)
Supplement: Supplementary file 1 [file ijms-24-03780-s001.zip › ijms-2193266-supplementary.pdf]

International Journal of *Molecular Sciences*

MDPI

Supplementary material

Comparative study of the effect of doping $ZnTiO_3$ with rare earths (La and Ce) on the adsorption and photodegradation of cyanide in aqueous systems

Ximena Jaramillo-Fierro [1,*], Guisella Cuenca [2], and John Ramón [2]

[1] Departamento de Química, Facultad de Ciencias Exactas y Naturales, Universidad Técnica Particular de Loja, San Cayetano Alto, Loja 1101608, Ecuador
[2] Ingeniería Química, Facultad de Ciencias Exactas y Naturales, Universidad Técnica Particular de Loja, San Cayetano Alto, Loja 1101608, Ecuador
* Correspondence: xvjaramillo@utpl.edu.ec; Tel.: +593-7-3701444

Abstract: Cyanide is a highly toxic compound that can pose serious health problems to both humans and aquatic organisms. Therefore, the present comparative study focuses on the removal of total cyanide from aqueous solutions by photocatalytic adsorption and degradation methods using $ZnTiO_3$ (ZTO), La/$ZnTiO_3$ (La/ZTO) and Ce/$ZnTiO_3$ (Ce/ZTO). The nanoparticles were synthesized by the sol-gel method and characterized by XRD, SEM, EDS, DRS, and BET. The adsorption equilibrium data were fitted to the Langmuir and Freundlich isotherm models. Adsorption kinetics were also evaluated using the pseudo-first order and pseudo-second order models and the intraparticle diffusion model. Likewise, the photodegradation of cyanide under simulated sunlight was investigated and the reusability of the synthesized nanoparticles for cyanide removal in aqueous systems was determined. The results demonstrated the effectiveness of doping with La and Ce to improve the adsorbent and photocatalytic properties of ZTO. In general, La/ZTO showed the maximum percentage of total cyanide removal (99.0%) followed by Ce/ZTO (97.0%) and ZTO (93.6%). Finally, based on the evidence of this study, a mechanism for the removal of total cyanide from aqueous solutions using the synthesized nanoparticles was proposed.

Keywords: Adsorption; Photocatalysis; Cyanide; Zinc Titanate; Lanthanides; Nanoparticles

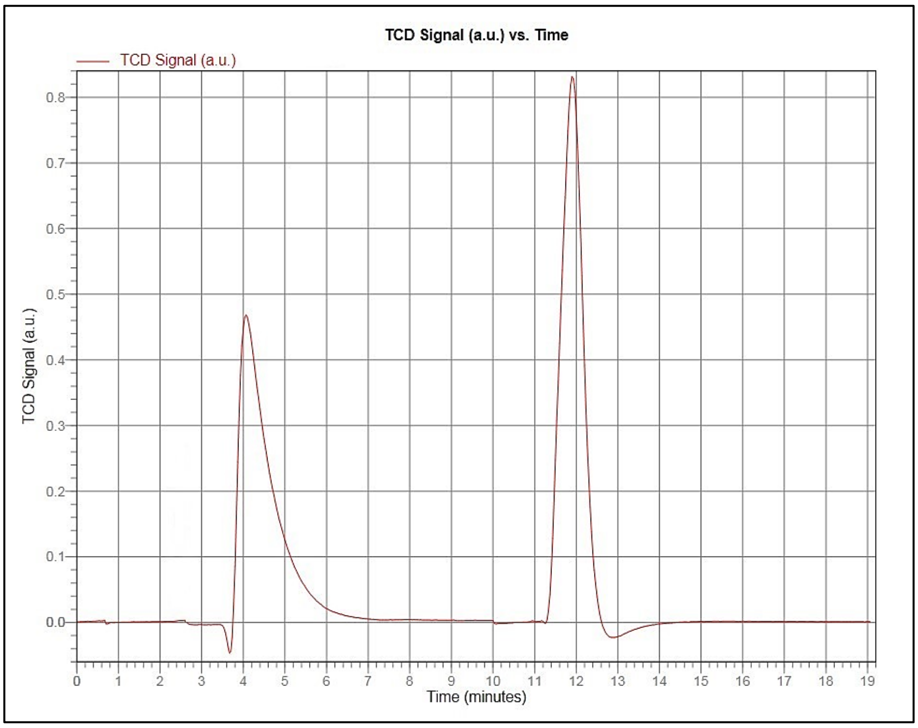
TCD Signal (a.u.) vs. Time
TCD Signal (a.u.)
TCD Signal (a.u.)
Time (minutes)
0.8
0.7
0.6
0.5
0.4
0.3
0.2
0.1
0.0
0 1 2 3 4 5 6 7 8 9 10 11 12 13 14 15 16 17 18 19

(a)

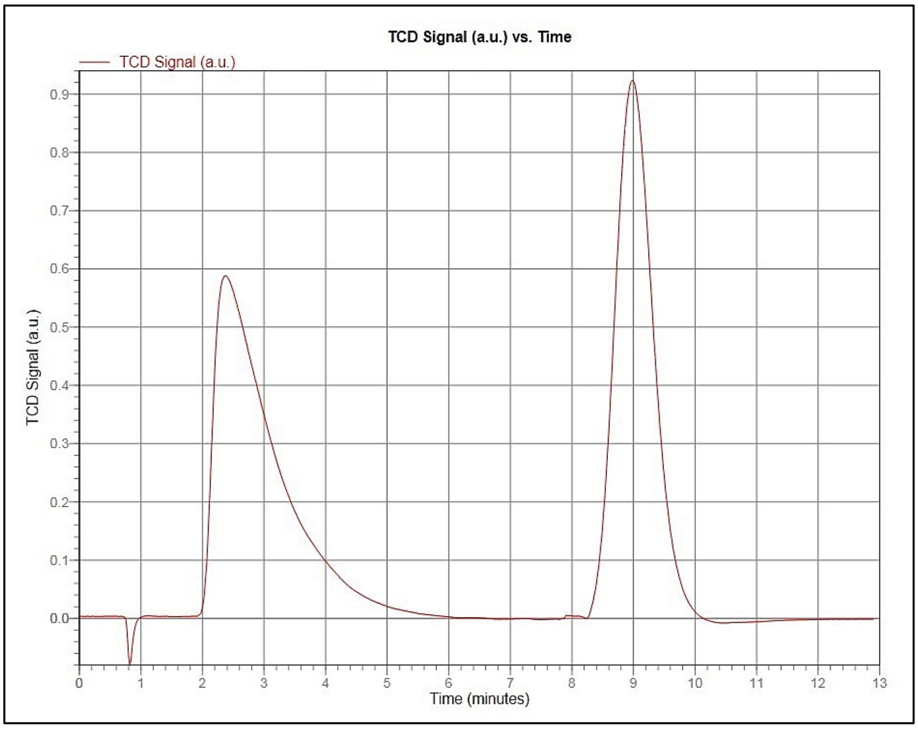
TCD Signal (a.u.) vs. Time
TCD Signal (a.u.)
TCD Signal (a.u.)
Time (minutes)
0.9
0.8
0.7
0.6
0.5
0.4
0.3
0.2
0.1
0.0
0 1 2 3 4 5 6 7 8 9 10 11 12 13

(b)

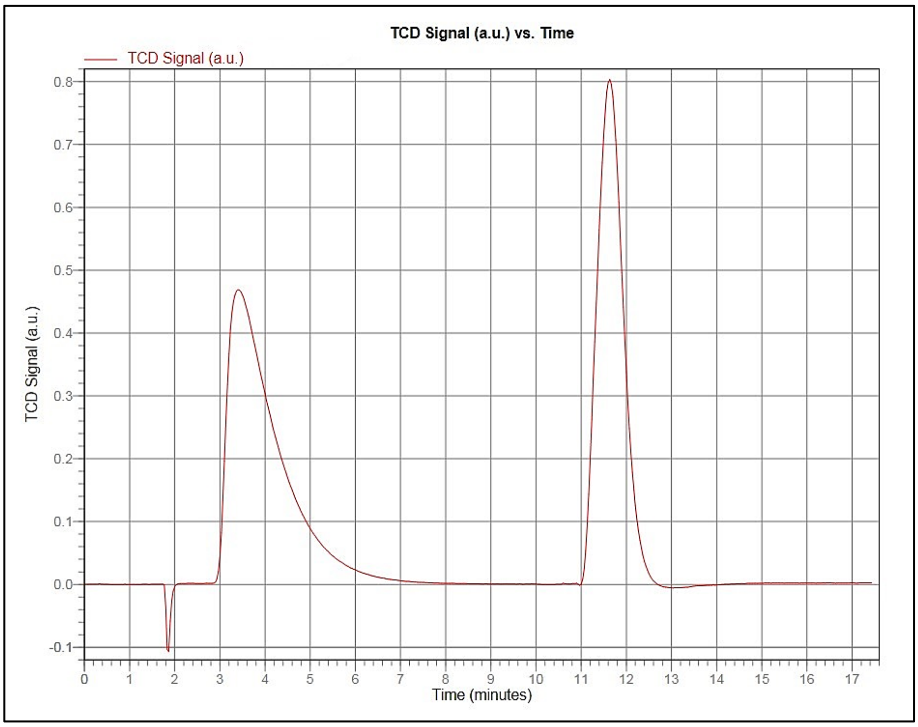

(c)

Figure S1. Nitrogen adsorption-desorption curves for SSA (m^2 g^{-1}) of **(a)** ZTO, **(b)** La/ZTO, and **(c)** Ce/ZTO by the single point method using the BET equation.
